# Supplementary material for: MetaRibo-Seq measures translation in microbiomes
Source: Nat Commun. 2020 Jun 29;11:3268. doi: 10.1038/s41467-020-17081-z (PMC7324362; doi:10.1038/s41467-020-17081-z)
Supplement: Supplementary file 10 — Supplementary Data 7 [file 41467_2020_17081_MOESM10_ESM.zip › File2/Confidence_High_Taxonomy/386804_out.krona.html]

Javascript must be enabled to view this page.

members
magnitude
magnitudeUnassigned
count
unassigned
taxon
rank

386804\_out

189

189
2
superkingdom

phylum
189
976

class
200643
189

order
189
171549

family
171550
187

genus
187
2

SRS050422\_contig\_number\_contig-100\_3878.3878SRS100907\_contig\_number\_contig-100\_14722.14723
239759

species
328814
1

SRS013098\_contig\_number\_15960


SRS042628\_contig\_number\_13036SRS050998\_contig\_number\_5059SRS098655\_contig\_number\_9964
3
28117
species

328813
1

SRS051882\_contig\_number\_12599
species

species
1946009
1

SRS098717\_contig\_number\_4448

species

SRS018541\_contig\_number\_2826SRS1041157\_contig\_number\_contig-100\_2516.36887SRS140492\_contig\_number\_21596
3
1118061


SRS046369\_contig\_number\_50
1
1872444
species

species

SRS013215\_contig\_number\_343SRS014855\_contig\_number\_3827SRS016018\_contig\_number\_1659SRS019161\_contig\_number\_34165SRS019286\_contig\_number\_4441SRS019968\_contig\_number\_2628SRS020233\_contig\_number\_35124SRS048870\_contig\_number\_20350SRS053398\_contig\_number\_24497SRS074964\_contig\_number\_4085SRS098644\_contig\_number\_48223SRS1041033\_contig\_number\_20297SRS104636\_contig\_number\_29468SRS1054716\_contig\_number\_10366SRS1054928\_contig\_number\_3720SRS147766\_contig\_number\_contig-100\_3982.126127
16
1262694


SRS101376\_contig\_number\_3690
1
1896970
species


SRS024388\_contig\_number\_9987SRS077024\_contig\_number\_3940
2
1263035
species

species
2

SRS045004\_contig\_number\_12053SRS143342\_contig\_number\_4454
1896969

214856
154

SRS011302\_contig\_number\_27916SRS012273\_contig\_number\_774SRS013965\_contig\_number\_39988SRS014459\_contig\_number\_30981SRS014613\_contig\_number\_14886SRS014683\_contig\_number\_4321SRS014948\_contig\_number\_13219SRS014979\_contig\_number\_34358SRS015190\_contig\_number\_14742SRS015217\_contig\_number\_4106SRS015431\_contig\_number\_64671SRS015794\_contig\_number\_contig-100\_132.69269SRS015854\_contig\_number\_16634SRS015890\_contig\_number\_21969SRS016056\_contig\_number\_3209SRS016095\_contig\_number\_1053SRS016267\_contig\_number\_11371SRS016335\_contig\_number\_35272SRS016897\_contig\_number\_478SRS017103\_contig\_number\_7123SRS017191\_contig\_number\_18881SRS017247\_contig\_number\_3494SRS017521\_contig\_number\_49211SRS018313\_contig\_number\_11514SRS018351\_contig\_number\_10630SRS018427\_contig\_number\_14972SRS018575\_contig\_number\_contig-100\_1568.61035SRS018623\_contig\_number\_3503SRS018656\_contig\_number\_2627SRS018817\_contig\_number\_13509SRS018836\_contig\_number\_23660SRS018888\_contig\_number\_3529SRS018936\_contig\_number\_6583SRS018984\_contig\_number\_32677SRS019030\_contig\_number\_13231SRS019068\_contig\_number\_66322SRS019178\_contig\_number\_2194SRS019267\_contig\_number\_contig-100\_2722.55536SRS019601\_contig\_number\_7003SRS019787\_contig\_number\_2783SRS020394\_contig\_number\_12098SRS021948\_contig\_number\_29545SRS022071\_contig\_number\_2856SRS024075\_contig\_number\_12122SRS024132\_contig\_number\_contig-100\_13176.217752SRS024331\_contig\_number\_3080SRS024492\_contig\_number\_14740SRS024549\_contig\_number\_4312SRS042284\_contig\_number\_5375SRS043411\_contig\_number\_13068SRS043701\_contig\_number\_14861SRS043768\_contig\_number\_25017SRS045645\_contig\_number\_contig-100\_3648.172194SRS045826\_contig\_number\_15481SRS046717\_contig\_number\_2553SRS047014\_contig\_number\_contig-100\_20308.200508SRS049402\_contig\_number\_3657SRS049773\_contig\_number\_721SRS049900\_contig\_number\_17549SRS049959\_contig\_number\_13978SRS050925\_contig\_number\_24635SRS051610\_contig\_number\_7464SRS052697\_contig\_number\_9451SRS053214\_contig\_number\_2090SRS053335\_contig\_number\_17670SRS053649\_contig\_number\_12353SRS054590\_contig\_number\_1615SRS054905\_contig\_number\_19728SRS054956\_contig\_number\_contig-100\_2775.78365SRS056519\_contig\_number\_16336SRS057480\_contig\_number\_8998SRS058070\_contig\_number\_13259SRS058723\_contig\_number\_7887SRS063040\_contig\_number\_35348SRS063489\_contig\_number\_4591SRS063518\_contig\_number\_36804SRS064232\_contig\_number\_86SRS064276\_contig\_number\_18198SRS064645\_contig\_number\_810SRS064757\_contig\_number\_21341SRS065176\_contig\_number\_14257SRS065397\_contig\_number\_11969SRS075341\_contig\_number\_11998SRS075773\_contig\_number\_32207SRS075984\_contig\_number\_14598SRS077086\_contig\_number\_6314SRS077231\_contig\_number\_8023SRS077335\_contig\_number\_6338SRS077552\_contig\_number\_16761SRS077641\_contig\_number\_21825SRS077730\_contig\_number\_12858SRS078242\_contig\_number\_13597SRS078419\_contig\_number\_22274SRS097920\_contig\_number\_25153SRS098827\_contig\_number\_2608SRS098881\_contig\_number\_2544SRS100021\_contig\_number\_10940SRS103987\_contig\_number\_7338SRS1041032\_contig\_number\_5287SRS1041039\_contig\_number\_contig-100\_5496.135017SRS1041129\_contig\_number\_1890SRS1041132\_contig\_number\_8088SRS1041137\_contig\_number\_15144SRS1041142\_contig\_number\_1470SRS1041145\_contig\_number\_31895SRS1041164\_contig\_number\_3354SRS104327\_contig\_number\_contig-100\_2320.38892SRS104400\_contig\_number\_1410SRS104912\_contig\_number\_contig-100\_1892.49568SRS104975\_contig\_number\_24456SRS1055034\_contig\_number\_9829SRS1055056\_contig\_number\_12520SRS1055076\_contig\_number\_contig-100\_703.89992SRS1055083\_contig\_number\_4446SRS140513\_contig\_number\_13423SRS142503\_contig\_number\_3922SRS142542\_contig\_number\_3594SRS142712\_contig\_number\_1421SRS142890\_contig\_number\_22486SRS143085\_contig\_number\_3934SRS143417\_contig\_number\_19279SRS143466\_contig\_number\_17152SRS143523\_contig\_number\_22359SRS143722\_contig\_number\_15517SRS143876\_contig\_number\_23354SRS143895\_contig\_number\_19285SRS143991\_contig\_number\_1003SRS144183\_contig\_number\_34938SRS144362\_contig\_number\_21704SRS144506\_contig\_number\_27825SRS144537\_contig\_number\_5003SRS144603\_contig\_number\_16905SRS146764\_contig\_number\_10513SRS146813\_contig\_number\_9724SRS147039\_contig\_number\_contig-100\_2065.2066SRS147346\_contig\_number\_4367SRS147557\_contig\_number\_31288SRS147653\_contig\_number\_5754SRS147919\_contig\_number\_13494SRS148196\_contig\_number\_3409SRS148253\_contig\_number\_13161SRS148424\_contig\_number\_contig-100\_2441.163986SRS148511\_contig\_number\_15892SRS148784\_contig\_number\_contig-100\_1953.166465SRS148817\_contig\_number\_9382SRS148970\_contig\_number\_3229SRS150029\_contig\_number\_contig-100\_2123.123272SRS892882\_contig\_number\_12781SRS893300\_contig\_number\_660SRS893327\_contig\_number\_2009SRS893341\_contig\_number\_10728SRS893373\_contig\_number\_contig-100\_1865.84258SRS893382\_contig\_number\_contig-100\_1357.47136SRS971276\_contig\_number\_contig-100\_4024.194896
species

family
2005525
1

1
375288
genus

species
46503

SRS148721\_contig\_number\_32342
1

family
815
1

genus
816
1

246787

SRS065504\_contig\_number\_30860
1
species
